# Chromatin binding and silencing: Two roles of the same protein Lem2

**DOI:** 10.15698/mic2016.04.495

**Published:** 2016-04-04

**Authors:** Ramón Ramos Barrales, Sigurd Braun

**Affiliations:** 1 Department of Physiological Chemistry, Biomedical Center, Ludwig-Maximilians-University of Munich, Grosshaderner Str. 9, 82152 Martinsried, Germany.

**Keywords:** heterochromatin, perinuclear silencing, tethering, LEM, lamin-associated proteins

## Abstract

Transcriptionally repressed chromatin localizes to specific areas within the eukaryotic nucleus and is often found at the nuclear periphery, which is thought to provide a specialized compartment for gene silencing. However, the molecular mechanisms that establish this spatial chromatin organization are still poorly understood. In our recent work (Barrales *et al.* 2016), we identified the nuclear envelope protein Lem2, a homolog of metazoan lamin-associated proteins (LAPs), as a relevant factor for heterochromatin silencing and perinuclear localization in the fission yeast *Schizosaccharomyces pombe*. Several other LAPs have previously been reported to associate with heterochromatin, and it has been proposed that this interaction might directly contribute to gene repression, perhaps through tethering via chromatin-binding domains like the LEM domain. We demonstrated that the LEM domain of Lem2 is indeed essential for centromere binding and perinuclear tethering. However, we made the surprising finding that tethering via the LEM domain is functionally independent of Lem2’s role in silencing, which instead is mediated by a different part of the protein, the MSC domain. Our study demonstrates that tethering and silencing, although mediated by the same molecule, Lem2, can be mechanistically separated. This further unveils a complex function of this protein at the interface between the nuclear periphery and silent chromatin, which might be preserved among the other members of this conserved family of LEM proteins.

Chromatin is non-randomly distributed inside the eukaryotic nucleus. Transcriptionally active euchromatin localizes to the interior, whereas silent heterochromatin is frequently found at the nuclear periphery. This type of repressed chromatin is in direct contact with the nuclear lamina, a meshwork of intermediate filaments called lamins that are attached to integral proteins of the inner nuclear membrane, the lamina-associated proteins (LAPs). Many of these LAPs contain protein domains that bind to DNA or chromatin. For example, members of the family of LEM (LAP2-Emerin-MAN1) domain containing proteins interact with so-called barrier-to-autointegration factors (BAFs), which are non-sequence-specific DNA-binding factors. The chromosomal regions that associate with the nuclear lamina are highly enriched for poorly expressed genes and repressive chromatin modifications. However, despite this striking correlation, it remains still elusive whether the interaction with lamins or LAPs contributes to the repressed state of heterochromatin. In particular, the large number of metazoan LAPs and their potential redundancy makes it very challenging to test their functional relevance in gene repression. Thus, studying their roles in model systems with less complexity provides an expedient alternative.

The fission yeast *S. pombe* is an outstanding model organism for studying heterochromatin formation and nuclear organization. In contrast to its distant relative *Saccharomyces cerevisiae* (budding or bakery yeast), many of the hallmarks of heterochromatin found in higher eukaryotes (repressive histone H3K9 methylation, HP1 proteins, RNA interference) are conserved in *S. pombe*. This yeast species also has perinuclear heterochromatin: The three centromeres localizes together at the nuclear membrane next to the spindle pole body (the yeast equivalent of the metazoan centrosome), while the six telomeres form 2-3 clusters at the opposite nuclear periphery. Importantly, although lamins are absent in yeast, a small number of LAP homologs have been identified: two LEM domain-containing proteins, Lem2 and Man1, and another nuclear membrane protein, Ima1, which shows homology to mammalian Samp1/NET5. This low redundancy makes *S. pombe* a perfect model organism to analyze the implication of these proteins in chromatin localization and silencing.

Through a genetic screen, we identified Lem2 as a novel silencing factor in *S. pombe*. Loss of *lem2+* causes de-repression of all constitutive heterochromatic domains (centromeres, telomeres and mating type locus), while deletion of the other LAP homologs, Man1 or Ima1, does not alleviate silencing, neither alone nor in combination with *lem2*∆. This finding indicates that Lem2 is the only LAP homolog involved in silencing in *S. pombe*. Nonetheless, silent chromatin is not completely de-repressed in absence of Lem2 and the phenotype is significantly weaker than seen for cells lacking the sole H3K9 methyltransferase Clr4, which is essential for heterochromatin maintenance. This finding suggested that Lem2 might act redundantly with other pathways in controlling silencing of heterochromatin. To test this hypothesis, we took a functional genomics approach by performing a Synthetic Genetic Array (SGA) screen: We crossed *lem2*∆ with a mutant library of non-essential genes and systematically examined this genome-wide collection of double mutants for synthetic silencing defects. This approach revealed indeed many genes that act redundantly with *lem2+*. Notably, several of these genes encode other nuclear membrane-associated factors. Quantitative examination of the silencing defects allowed us dissecting the redundant pathways. While pericentromeric silencing mainly requires Lem2 and the RNAi machinery, the full repression of subtelomeric genes requires additional factors including the telomere-associated specific protein Taz1. Thus, Lem2 is part of a complex network of silencing factors that cooperate at the nuclear periphery to ensure efficient and specific gene silencing. At the molecular level, we found that Lem2 controls the recruitment of antagonistic silencing factors to heterochromatin. Loss of Lem2 causes decreased binding of the Snf2-like/HDAC repressor complex SHREC, while the binding of its competitor, the anti-silencing factor Epe1, is increased. Intriguingly, deleting *epe1+* restores silencing in the absence of Lem2 at various heterochromatic domains, suggesting that the augmented chromatin level of Epe1 is the main reason for the silencing defect at these chromatin regions.

In *S. pombe*, all major heterochromatic regions localize to the nuclear periphery in close proximity to the nuclear envelope. While Lem2 was previously shown to affect telomere anchoring, a contribution to centromere localization was not observed. Yet, the SGA analysis revealed various factors that not only act redundantly but also show a similar localization to Lem2 (i.e. a nuclear envelope localization with accumulation at the spindle pole body). Among these redundant factors was Csi1, a factor involved in centromere clustering. We therefore tested whether these two factors, Lem2 and Csi1, also cooperate at the level of centromere localization. Indeed, we found that about 20% of *lem2*∆ *csi1*∆ cells display a complete delocalization of all three centromeres from the nuclear membrane — a phenotype that is neither seen in the *lem2*∆ nor *csi1*∆ single mutant (Figure 1). Remarkably, we also observed a synthetic defect for telomere anchoring in cells deficient for Lem2 and RNAi. Thus, the redundant function of Lem2 in silencing is reflected by its redundant role in controlling heterochromatin localization. This prompted us to study whether silencing by Lem2 is primarily a consequence of its function in tethering heterochromatin to the periphery.

**Figure 1:**
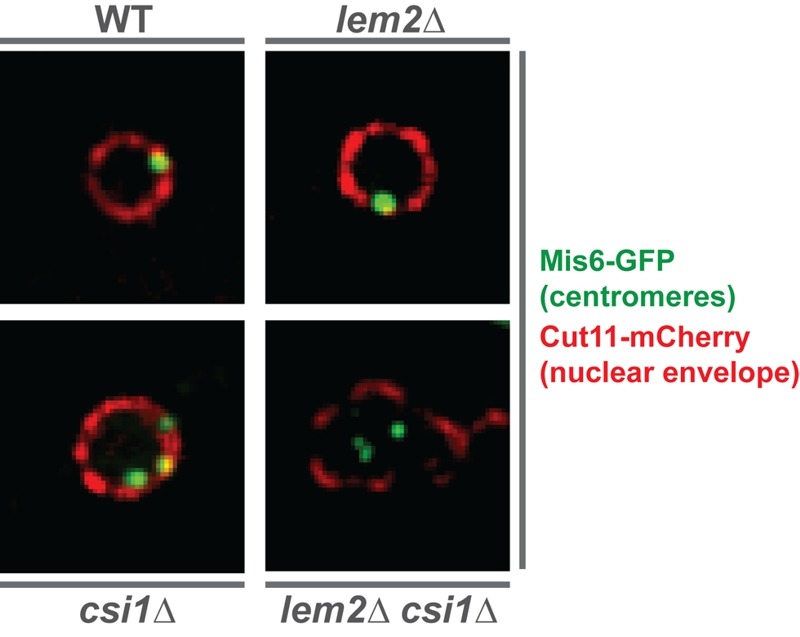
FIGURE 1: Centromeres delocalize from the nuclear periphery in the double mutant *lem2*∆ *csi1*∆. Images show the relative localization of centromeres (green) with respect to the nuclear envelope (red) in WT and indicated mutant nucleus. The kinetochore protein Mis6 is tagged with GFP for visualizing centromeres, while the nuclear pore protein Cut11 is fused to mCherry for detecting the nuclear envelope.

Lem2 contains two domains at its N- and C-terminus, the conserved LEM and MSC (MAN1-Src1 C-terminal) domains, respectively, which are separated by two transmembrane domains. Both nucleoplasmic domains have been proposed to mediate interaction with DNA and/or chromatin. In order to examine which of these domains are required for heterochromatin localization and silencing, we generated truncated versions of Lem2 harbouring only the LEM or MSC domain (including the two transmembrane domains). These constructs were expressed in a *lem2*∆ strain to test for complementation of the lem2-specific phenotypes. Notably, we found that Lem2 associates specifically with centromeric chromatin and that the N-terminal part containing the LEM domain is necessary and sufficient for this chromatin interaction. Moreover, we demonstrated that the LEM domain is also required for the perinuclear localization of centromeres, providing experimental evidence of the previous notion that the LEM domain mediates chromatin association and peripheral recruitment *in vivo*. However, when examining silencing we surprisingly found the exact opposite requirement: not the LEM but the MSC domain is necessary for silencing of all heterochromatic domains. Interestingly, this requirement of the MSC domain was also observed for telomere anchoring, indicating that the mechanisms of centromere and telomere recruitment are substantially different. Nevertheless, although the MSC domain is not required for interaction of Lem2 with chromatin, its presence at the nuclear envelope is a prerequisite for its proper function in silencing. Thus, rather than tethering chromatin to the periphery, the MSC domain might be involved in the recruitment and concentration of critical factors to peripheral heterochromatin.

In summary, we uncovered a complex function of the conserved inner nuclear membrane protein Lem2, which mediates both chromatin localization and silencing yet by utilizing different functional domains (Figure 2): The MSC domain is the only domain required for silencing and also has a function in telomere localization. Conversely, the LEM domain contributes to centromere binding and localization. Until recently, most of the studies on LAPs have been focusing on the LEM domain assuming that this domain is critical for silencing due to its role in chromatin interaction. However, here we unveiled an unanticipated role of the MSC domain and demonstrate its importance for transcriptional silencing. Thus, this study adds a new perspective on the role of LAPs in chromatin silencing and localization. It further demonstrates the significance of *S. pombe* as a model to study the role of this conserved family of LEM domain proteins at the molecular level, increasing the understanding of their implications in development and disease.

**Figure 2:**
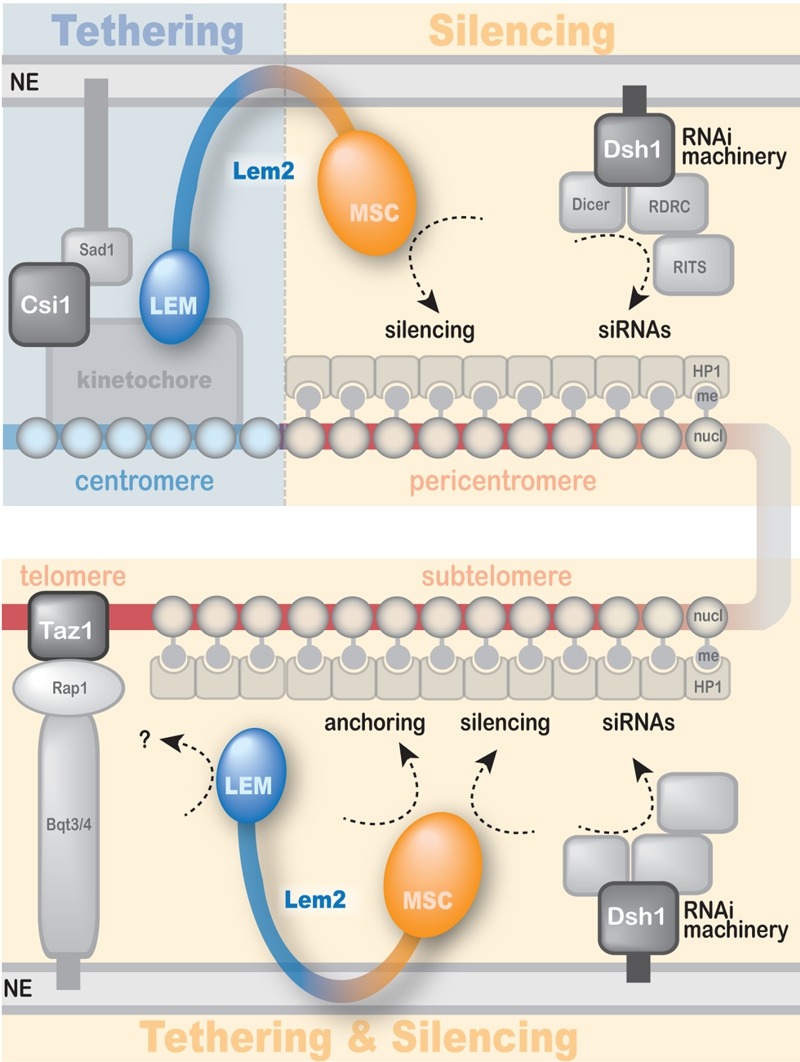
FIGURE 2: A model for Lem2 function in heterochromatin localization and silencing. Lem2 mediates anchoring to the nuclear periphery of centromeres and telomeres through its LEM domain and MSC domain, respectively. Silencing of both heterochromatin domains is mediated exclusively through its MSC domain. Both functions cooperate with other redundant pathways (i.e. Csi1, RNAi, Taz1). See text for details.

